# Subventricular zone progenitors in time and space: generating neuronal diversity

**DOI:** 10.3389/fncel.2014.00434

**Published:** 2014-12-22

**Authors:** Eduardo B. Sequerra

**Affiliations:** Instituto do Cérebro, Universidade Federal do Rio Grande do NorteNatal, RN, Brazil

**Keywords:** heterogeneity, interneuron sub-type, morphogens, transcription factors, cellular differentiation

## Abstract

The adult mammalian brain harbors a population of cells around their lateral ventricles capable of giving rise to new neurons throughout life. The so-called subventricular zone (SVZ) is a heterogeneous germinative niche in regard to the neuronal types it generates. SVZ progenitors give rise to different olfactory bulb (OB) interneuron types in accordance to their position along the ventricles. Here, I review data showing the difference between progenitors located along different parts of the SVZ axes and ages. I also discuss possible mechanisms for the origin of this diversity.

## SVZ progeny in space and time

**Figure 1:**
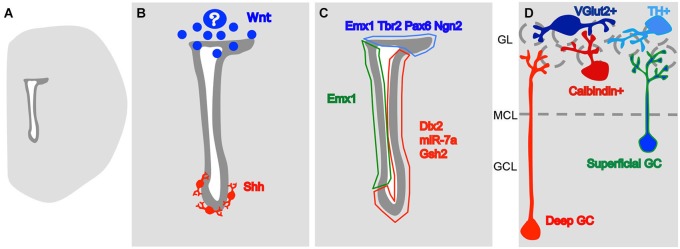
**A model for the origin of heterogeneity in the SVZ. (A)** Drawing of a coronal slice of one hemisphere, representing the lateral ventricle (in white) and the SVZ (in dark gray). **(B)** The morphogens so far described to influence the generation of different OB interneuron subtypes. Wnt is produced dorsally, by an unknown source, and Shh is produced by neurons at the vicinity of the ventral SVZ. **(C)** These signaling molecules probably lead to differential expression of type-specific gene regulators dorsally (in blue) on the lateral wall, close to the striatum (in red) and on the medial wall, close to the septum (in green). **(D)** The expression of these regulators leads to the generation of OB interneuron type in a space-segregated manner. Neurons in blue are generated dorsally while the neurons in red come from ventral positions and the type in green and blue come both from the medial and dorsal SVZs. GCL- granular cell layer, MCL- mitral cell layer (represented by a dashed line), GL- glomerular layer (the glomeruli are represented by dashed circles).

The SVZ is one of the main neural stem cell niches in the adult mammalian brain. SVZ progenitors continuously give rise to new neurons that migrate to and differentiate in the ipsilateral olfactory bulb (OB; Altman, 1969; Luskin, 1993; Lois and Alvarez-Buylla, 1994). Once there, newly generated neurons differentiate into multiple neuronal types that participate in the OB local circuitry (Luskin, 1993; Brill et al., 2009; Merkle et al., 2014). Progenitors located at different positions of the antero-posterior and dorso-ventral axes of the lateral ventricles originate these neuronal types in a spatial-segregated manner (Figure 1; Merkle et al., 2007). However, it is still debated to which extent this co-relation between progenitor position and neuronal type generated is due to an internal program or distinct environmental factors impinging onto progenitors (for discussion see, Sequerra et al., 2013).

The first evidence supporting that the SVZ is indeed a heterogeneous territory came from a series of studies performed at Marla Luskin’s lab. This group described that neuroblasts migrating to the OB proliferate (Menezes et al., 1995). However, many of them leave the cell cycle along the pathway forming a posterior to anterior gradient of proliferation along the RMS (Coskun and Luskin, 2001). The progenitors located along different points of dorso-ventral and antero-posterior axes of the ventricular wall display different cell cycle kinetics and cell type density (Falcão et al., 2012). Retroviral labeling of the proliferative progenitors in the anterior SVZ (Luskin, 1993) or at the horizontal limb of the rostral migratory stream (Smith and Luskin, 1998), demonstrated that progenitors of granular neurons leave the cell cycle earlier (more posteriorly) than the ones that will generate the periglomerular (PG) neurons. These data, later reproduced by other group (Hack et al., 2005), show that different populations of newly generated OB neurons segregate very early, while still migrating. But how early would such segregation take place?

During the embryonic development of the nervous system, different neuronal types are generated in morphogenetic territories that are formed through the establishment of gradients of diffusive molecules and the subsequent expression of type-specific transcription factors (TFs; Jessell, 2000). In the embryonic telencephalon, different types of neurons are generated according to their position in the dorso-ventral axis: cholinergic in the ventral ventricular zone (VZ), GABAergic in the ventro-lateral and glutamatergic in the dorsal (Marín and Rubenstein, 2001). Therefore, a question to be addressed was whether the spatial segregation of the SVZ germinative niche could represent a continuum of that in the embryonic telencephalon. Since the adult neural stem cells of the SVZ are directly derived from radial glia cells (Alves et al., 2002; Tramontin et al., 2003; Merkle et al., 2004), it is possible to label their radial process at different pial locations during early postnatal stages and then track the progeny of the generated SVZ progenitors (Merkle et al., 2007; Ventura and Goldman, 2007). Surprisingly for that time, the dorsal radial glia, that during embryogenesis gives rise to glutamatergic cortical neurons (Schuurmans et al., 2004), was shown to contribute inhibitory OB interneurons after embryogenesis (Merkle et al., 2007; Ventura and Goldman, 2007), and the progeny of radial glia located at different positions of the lateral ventricle dorso-ventral and antero-posterior axes give rise to different OB interneuron subtypes (Figure 1; Kelsch et al., 2007; Merkle et al., 2007; Paez-Gonzalez et al., 2014). Collectively, these data indicate that the adult ventricular wall is also divided in morphogenetic territories specialized in forming specific neuronal types.

Interestingly, different OB interneuron types are not only generated in a spatially segregated manner but also in a different time dynamics. De Marchis et al. (2007) injected Fluorogold in the anterior SVZ of neonatal and adult mice and described their progeny according to the neuronal types generated in the glomerular layer (GL). Batista-Brito et al. (2008) analyzed mice that had an inducible Cre-recombinase regulated by the Dlx1/2 enhancer, which is expressed in SVZ type C cells, in different time points, from embryogenesis to postnatal and adults. Li et al. (2011) analyzed the identity of new neurons labeled with BrdU at different ages. All three groups found that different OB interneuron types are generated in very specific time patterns, some exclusively during embryogenesis and first postnatal week, like the Parvalbumin^+^ cells of the external plexiform layer (EPL; Batista-Brito et al., 2008; Li et al., 2011), but others keep their production consistently during life, like the granular and PG Calretinin^+^ cells (De Marchis et al., 2007; Batista-Brito et al., 2008; Li et al., 2011). Although most of the Tyrosine hydroylase (TH)^+^ PG neurons are generated embryonically and during the first postnatal week (Li et al., 2011), there are contradictory data in respect to the tendency of adult-generated neurons to give rise to this OB subtype. While Fluorogold (De Marchis et al., 2007) and BrdU injections (Whitman and Greer, 2007) suggest that a higher percentage of neurons born in the adult differentiate into TH^+^ PG neurons, lineage analysis of the inducible Dlx1/2 enhancer progeny indicates that the percentage of cells that choose this type declines from embryogenesis to P30 (Batista-Brito et al., 2008). Independent of this contradiction, these data collectively show that the multiple types of OB subtypes are generated in a time-dependent fashion, suggesting dynamics in the activity of the SVZ during life. Since the different types are generated in different spatial domains it will be interesting to see if these domains are differentially regulated during time or if they can even fluctuate in size.

Different granular neuron subtypes are also mostly generated in segregated periods. The administration of BrdU or retroviral injections in the SVZ during the first postnatal week reveals that during this period superficial granular neurons are generated, whereas neurons generated in adults assume preferentially a deeper position (in respect to their position in the granular layer; Lemasson et al., 2005; Kelsch et al., 2007).

Although the main populations produced by the adult SVZ are granular or PG neurons (Luskin, 1993; Lois and Alvarez-Buylla, 1994), this region also produces other interneuron populations in a smaller number. The EPL has a much lower cell density than the granule cell layer (GCL) and GL. However, the EPL interneurons are generated at the SVZ with a peek during the first postnatal days but continuously along adulthood (Winner et al., 2002; Batista-Brito et al., 2008; Yang, 2008; Li et al., 2011). Merkle et al. (2014) recently described populations of EPL and mitral cell layer interneurons that are generated in the ventral SVZ during adulthood.

Through the analysis of the progeny of Neurog2^+^ progenitors, Brill et al. (2009) described the addition of a glutamatergic population to the PG population. PG neurons derived from these progenitors do not express Calretinin, TH, Sp8 or Calbindin and express the Vesicular glutamate transporter 2 (VGlut2; Brill et al., 2009). The morphologies of PG neurons from Neurog2^+^ lineage also vary over time. While neurons generated at embryonic stages project into the glomeruli, the posnatally-generated ones keep their dendrites around these structures (Winpenny et al., 2011). The adult-generated cells project to two or three glomeruli (Brill et al., 2009), a morphology typical of short-axon cells (Pinching and Powell, 1971). The neurochemical identity of short-axon neurons though, is currently under check. These cells were firstly described as glutamatergic since they do not have an active GAD_65_ promoter and the interglomerular transmission, attributed to these cells based on their morphology, is inhibited by ionotrophic glutamate receptors antagonists (Aungst et al., 2003). However, further histological (Kosaka and Kosaka, 2008; Kiyokage et al., 2010) and more careful functional analysis of their projection to the external tufted cells (Liu et al., 2013), show that interglomerular projecting neurons are actually GABAergic and dopaminergic instead of glutamatergic. The large soma TH^+^ cells in the GL, presumably short axon cells, are generated during embryogenesis and first postnatal week (Kosaka and Kosaka, 2009) and some of these neurons, co-expressing Parvalbumin at the border between the EPL and the GL, are generated in the postnatal but not the adult SVZ (Yang, 2008). Therefore, it remains unclear the role of the VGlut2^+^, with short axon cell-like morphology, interneurons in the adult OB circuitry.

The segregation of the SVZ into multiple progenitor domains and windows leads us to ask what are the mechanisms involved in the generation of this diversity. For having some insights about this mostly unanswered question, I will review the existence of signaling pathways in the SVZ that can regulate the formation of domains for subtype-specific neurogenesis. I will also review the gene transcription regulators that have a regionally restricted pattern of expression and, therefore, can be downstream of these signaling pathways.

## Establishment of morphogenetic territories in the SVZ

Being the SVZ a heterogeneous territory in respect to the neuronal populations it produces, how are these spatial differences formed? As said above, the embryonic ventricular cells are divided in morphogenetic territories and as the adult SVZ derives from it, the information can be passed on as an internal program. Alternatively, new information can be added in the postnatal/adult milieu either maintaining the original program or overwriting it.

The ventral adult SVZ display an active Sonic Hedgehog (Shh) signaling (Figure 1B; Ihrie et al., 2011). The disruption of this signaling in the ventral SVZ leads to a shift of production of OB interneurons to dorsal phenotypes, superficial granular cells and TH^+^ PG neurons. The dorsal SVZ however, is irresponsive to Shh administration, unless a constitutively active Smo (a Shh receptor) is induced (Ihrie et al., 2011). Therefore, the adult morphogenetic environment constantly maintains the ventral SVZ territory. Although SVZ progenitors can respond to their environment, their receptor composition differs, and their plasticity is limited.

In opposition to ventral Shh, the dorsal SVZ displays Wnt signaling (Figure 1B; Azim et al., 2014). The activation of this signaling contributes to the activation of the dorsal TF Tbr2 (Azim et al., 2014). The knock down of the downstream players of Wnt/Planar cell polarity signaling, Dvl2 or Vangl2, leads to a specific decrease in the generation of superficial granular neurons (Hirota et al., 2012), that are typically generated in more dorsal parts of the SVZ (Figure 1D; Merkle et al., 2007). It is still not clear at what point interfering with Wnt signaling leads to the generation of ventrally-generated interneuron subtypes, however, it seems clear that dorsal TFs in the SVZ are positively affected by this molecule.

Therefore, the SVZ regionalization seems to be actively maintained during the adult life. Since the production of different interneuron subtypes is age-dependent it will be interesting to see how these signaling pathways fluctuate in time.

## The SVZ molecular landscape

The differential expression of morphogens along the SVZ axes leads to the next step in cell line segregation that is an internal cascade of molecular events that lead to their specification into an OB neuronal subtype. TFs and microRNAs are expressed in discrete regions of the SVZ niche (Figure 1C).

Neurog2, Tbr2 and Tbr1, which are involved in specification of VGlut2^+^ interneurons of the GL, are restricted to the dorsal part of the SVZ around the lateral ventricles (Brill et al., 2009). Neurog2 and Tbr2 are expressed by fast proliferating, type C cells while Tbr1 is expressed later in the lineage (Brill et al., 2009). Pax6, a TF that takes part both in TH^+^ PG neuron (Hack et al., 2005; Kohwi et al., 2005; Brill et al., 2008) and in superficial granular neuron specification (Kohwi et al., 2005), is mostly expressed by the dorsal SVZ and by few cells in the lateral SVZ (Brill et al., 2008; de Chevigny et al., 2012a). The Emx1 lineage, that gives rise to superficial granule neurons and preferentially gives rise to Calretinin^+^ interneurons, is restricted to the dorsal and septal SVZs (Kohwi et al., 2007; Young et al., 2007). Dlx2, which works in conjunction with Pax6 to specify dopaminergic PG neurons (Brill et al., 2008), is expressed mainly by cells of the lateral wall of the ventricle (where it has no clear role interneuron type specification) but the dorsally generated population turns it on after migrating as neuroblasts in the RMS (de Chevigny et al., 2012a). Pax6 restriction to the dorsal aspect of the SVZ is regulated by an opposing gradient of the micro-RNA miR-7a, inhibiting its translation. The inhibition of miR-7a expression leads to an increase in dopaminergic PG neuron production (de Chevigny et al., 2012b). The Gsh2 lineage is located at the lateral wall of the ventricle and seems to be the lineage that produces Calbindin^+^ PG neurons (Young et al., 2007). Merkle et al. (2014) identified a subpopulation of SVZ cells located at the anterior region of the ventral lateral ventricles, that expresses Nkx6.2 and gives rise to EPL and mitral cell layer interneurons. Other TFs were shown to be restricted to a region of the SVZ, like Nkx2.1 and Dbx1 (Young et al., 2007), but their contribution to the generation of the different subtypes is not available yet.

Therefore, the postnatal/adult SVZ seems to be compartmentalized. During development, the neuroepithelium gets divided in physical structures, called neuromeres, which differentially express adhesion molecules (Redies and Takeichi, 1996). Actually, EphB1, EphB2, ephrin-B and VCAM1 are expressed exclusively at the lateral wall of the SVZ (Conover et al., 2000; Kokovay et al., 2012) while EphB3 is expressed on the septal side (del Valle et al., 2011). It will be important in the future to test how the differential expression of these molecules restricts cell-cell communication and migration and, consequently, the access of different populations to region-specific signaling molecules. Mellitzer et al. (1999) have shown that ephrin signaling restricts communication through GAP junctions to cells in the same neuromere. The postnatal dorsal SVZ (Freitas et al., 2012) and the adult lateral wall (Lacar et al., 2011) have local nets of astrocytes connected by GAP junctions but it is not clear at what extent these nets connect between compartments at the borders. A definite proof of the existence of physical compartments is still missing.

## Relevance to human adult SVZ progenitor heterogeneity

Although the dynamics of OB interneuron subtype is very well described in mice, the relevance of this phenomenon for other species is starting to be understood. Of particular interest for medical sciences is the observation that in adult human OB there is little or no addition of new neurons (Sanai et al., 2011; Bergmann et al., 2012). However, for our discussion about the origin of the different OB interneuron subtypes, what is important is the source of these neurons. Is there a diversity of progenitors in the adult human brain capable of generating the OB interneuron pool? There is a massive reduction of neuroblast generation in the human lateral ventricles after the first year of life (Sanai et al., 2011) although the complete disappearance of neurogenesis in this region is controversial. There are groups that successfully detected neuronal markers, like PSA-NCAM, Dcx and and classIII-ßtubulin around the lateral ventricles (Curtis et al., 2007; Wang et al., 2011), on the olfactory tubercule (Wang et al., 2011) and around the olfactory ventricle (Curtis et al., 2007). Although these neuroblasts do not seem to be resulting in the addition of new neurons to the OB, they are possibly a source for the recently documented addition of interneurons to the human adult striatum (Ernst et al., 2014). There is not a current molecular analysis of the heterogeneity of neuronal progenitors in the human SVZ although we can expect it to be a common feature in primates. This idea is supported by the demonstration that both macaques (Tonchev et al., 2006) and marmosets (Azim et al., 2013) express type-specific TFs in the SVZ. Marmosets have a specific reduction of Tbr2^+^ progenitors from first days of life to adulthood (Azim et al., 2013). Therefore, although the species differences have to be investigated, the theoretical piece of information collected in experimentation with rodents can still be relevant for the better understanding of the human SVZ heterogeneity of neuronal precursors.

## Conclusion

The data published so far shows that the different neuronal lineages generated after birth to the OB are spatially and temporally segregated. Both morphogen signaling and molecular internal programs affect the specification of these cells.

Although we have some information on the internal programs of different interneuron subtypes and about their site and time of origin, little is known about how segregation between clones is kept. The criticisms lie around our technical limitations on testing the SVZ neural stem cells on their level of commitment. The argument supporting that clones in different locations are following an internal program is in dissonance with the observation that all the TFs cited above start to be expressed on type C cells or neuroblasts (for review, see Sequerra et al., 2013), not affecting the slow dividing stem cell on the top of the lineage. And the culture methods used so far for isolating stem cells, preferentially select fast dividing progenitors instead of the slow ones (for review, see Pastrana et al., 2011). Even the lack of response to Shh by dorsal progenitors detected by Ihrie et al. (2011) can be due to a lack of an inductive signal that makes these progenitors competent of responding to it (Waddington, 1940).

Although there are many similarities between the segregation of the embryonic and adult periventricular stem cell niches, there are important differences too. The adult brain is much bigger and less suitable for the establishment of molecular gradients. Instead, the adult SVZ has an intricate net of blood vessels (Mercier et al., 2002; Shen et al., 2008; Tavazoie et al., 2008; Snapyan et al., 2009) and axons (Höglinger et al., 2004, 2014; Paez-Gonzalez et al., 2014; Tong et al., 2014) bringing new players to the niche. Actually, the source of Shh to the ventral SVZ is composed of neurons located at the septum, preoptic nuclei and the stria terminalis, neurons that project into the SVZ (Figure 1B; Ihrie et al., 2011). The elimination of dopaminergic projections from the substantia nigra decreases proliferation in the SVZ (Baker et al., 2004) and it was recently shown that the substantia nigra and the ventral tegmented area project to distinct regions of the lateral ventricle wall (Höglinger et al., 2014). It is possible then, that activity in distinct brain regions differentially modulate SVZ progenitors depending on their location. Therefore, although some signaling pathways and molecular tools are re-used in the adult SVZ, the way they are played can be completely new, not seen in embryos.

There is clearly a lot to be done from now on. Many studies in the past considered the SVZ a homogeneous population. Molecules that were tested for general neurogenesis (for review, Lim and Alvarez-Buylla, 2014), for example, can be affecting a specific population and not others. New studies have to take into account the influence of signaling molecules to different SVZ populations, the differential expression of molecular determinants along the ventricle axes, and the consequences of changes in these dynamics to the generation of the different OB interneuron populations.

## Conflict of interest statement

The author declares that the research was conducted in the absence of any commercial or financial relationships that could be construed as a potential conflict of interest.
